# Diagnostic value of blood thiamine metabolites in Alzheimer’s disease examined by ^11^C-PiB PET scanning

**DOI:** 10.4155/fsoa-2016-0087

**Published:** 2017-02-23

**Authors:** Zhichun Chen, Xiaoli Pan, Guoqiang Fei, Shumei Pan, Weiqi Bao, Shuhua Ren, Yihui Guan, Chunjiu Zhong

**Affiliations:** 1 Department of Neurology, Zhongshan Hospital & Shanghai Medical College, State Key Laboratory of Medical Neurobiology, Institute of Brain Science & Collaborative Innovation Center for Brain Science, Fudan University, Shanghai 200032, China; 2 PET center, Huashan Hospital, Fudan University, Shanghai 200443, China

**Keywords:** Alzheimer’s disease, blood biomarker, diagnosis, PiB positive, PiB negative, positron emission tomography with ^11^C-Pittsburgh compound B, thiamine, thiamine diphosphate, thiamine metabolites, γ-value

## Abstract

**Aim:**

We evaluated the diagnostic value of blood thiamine metabolites for Alzheimer’s disease (AD) by using positron emission tomography with ^11^C-Pittsburgh compound B (^11^C-PiB PET) scanning.

**Methods:**

Thirty-eight clinically diagnosed AD patients were voluntarily recruited. Blood thiamine metabolites were measured by high-performance liquid chromatography. All the patients received ^11^C-PiB PET scanning for the measurement of cerebral amyloid deposition.

**Results:**

Thiamine diphosphate (TDP) had 66.7% sensitivity and 80.0% specificity for AD diagnosis, while the γ-value representing the best combination of thiamine metabolites and age had 24.2% sensitivity and 100.0% specificity according to the cut-off value of our previous study.

**Conclusion:**

Blood TDP but not γ-value exhibited results significant for AD diagnosis.

## Lay abstract

In this study, we investigated the diagnostic value of blood thiamine metabolites for Alzheimer’s disease as examined by positron emission tomography with ^11^C-Pittsburgh compound B scanning. Our results suggest that the reduction of blood thiamine diphosphate may be a stable biomarker useful for Alzheimer's disease diagnosis.

To date, we still lack practical peripheral biomarkers for the diagnosis of Alzheimer’s disease (AD). The guidelines for AD diagnosis published in 2011 recommended that existing biomarkers be used only for research, and not for regular clinical practice. These biomarkers include the reduction of β-amyloid 42 (Aβ_42_), elevations of phosphorylated tau and total tau in cerebrospinal fluid, glucose hypometabolism of temporal and parietal cortex assayed by ^18^F-fluorodeoxyglucose positron emission tomography (^18^F-FDG PET), Aβ deposition in brain cortices examined by ^11^C-Pittsburgh compound B (^11^C-PiB) PET and brain atrophy measured by MRI [1]. Tests for the existing biomarkers have significant disadvantages. Lumbar puncture for cerebrospinal fluid analysis is reliant on advanced clinical skills and may cause physical injury and pain, resulting in low patient compliance. ^11^C-PiB PET and ^18^F-FDG PET are expensive, and the diagnostic value of structural MRI is limited. Recently, some peripheral biomarkers have been assessed for AD diagnosis, including those from blood [2,3] and saliva [4,5]. In terms of blood biomarkers of AD, both Aβ oligomers and tau levels have been found to be useless for the prediction of AD development in recent studies [6,7]. Furthermore, plasma protein profiles, lipid profiles and inflammatory factors all have been reported as potential AD biomarkers [8–12]. However, because of the low repeatability and instability of these biomarkers tests, none of these are recommended for clinical AD diagnosis. This frustrating situation drives us to explore new peripheral biomarker(s) for AD diagnosis.

Our previous multicenter study demonstrated that blood thiamine metabolites detected by high-performance liquid chromatography (HPLC) exhibited significant diagnostic utility for AD [13]. Here, we determined to validate the diagnostic value of the measurement of fasting blood thiamine metabolites in AD patients combining with ^11^C-PiB PET scanning.

## Materials & methods

### Participants

This study was approved by the Committee on Medical Ethics of Zhongshan Hospital, Fudan University, Shanghai, China. Thirty-eight clinically diagnosed AD patients were recruited to voluntarily receive ^11^C-PiB PET scanning and the measurement of blood thiamine metabolites by HPLC from the Dementia Clinic of Neurological Department in Zhongshan hospital from October 2012 to November 2014. Informed consents were obtained from all AD patients or their caregivers. All of them took neuropsychological examinations, including the Mini-Mental Status Examination (MMSE), Clinical Dementia Rating (CDR), Activity of Daily Living (ADL) scales and Hamilton Depression Rating Scale. All patients also received cranial MRI and/or CT scans to exclude the possibilities of vascular dementia or occupied brain lesions. Blood folate, vitamin B_12_, fasting glucose and thyroid function were also examined. The diagnosis of AD was made by neurologists specialized in dementia according to the criteria of DSM IV and guideline for AD diagnosis published in 2011 (National Institute on Aging – Alzheimer’s Association workgroups) [1]. The inclusion criteria and exclusion criteria of AD subjects were the same as those listed in our previous study [13]. Different from the previous study [13], the current study also recruited patients with age of less than 65 years old. The demographic and clinical data of the current study are summarized in Table 1.

**Table 1.** **Demographic and clinical data of Alzheimer's disease subjects recruited in this study.**

**Name**	**Age**	**Sex**	**MMSE**	**ADL**	**CDR**	**^11^C-PiB (negative/positive)**	**TDP**	**TMP**	**Thiamine**	**γ-value**
Case 1	54	M	17	8	1	Negative	116.99	22.15	6.12	33.50
Case 2	63	M	20	8	1	Negative	86.48	17.29	2.90	55.94
Case 3	71	M	25	8	0.5	Negative	105.47	16.71	6.40	64.83
Case 4	80	M	17	11	1	Negative	149.35	9.86	1.62	49.11
Case 5	86	M	23	10	0.5	Negative	130.54	2.03	4.57	74.60
Case 6	48	M	17.5	11	0.5	Positive	103.29	3.99	1.79	26.05
Case 7	51	F	16	8	1	Positive	96.14	17.57	6.64	36.87
Case 8	51	F	23	9	1	Positive	90.05	8.82	4.59	37.61
Case 9	52	F	15	10	1	Positive	80.50	7.90	16.03	52.71
Case 10	54	F	11	8	1	Positive	49.48	37.08	0.00	56.83
Case 11	56	M	13	11	1	Positive	75.59	67.04	2.68	49.42
Case 12	57	M	17	9	1	Positive	142.92	11.07	3.50	28.49
Case 13	57	F	5	15	2	Positive	90.21	11.94	2.76	43.78
Case 14	58	M	22	9	0.5	Positive	80.55	8.82	1.33	47.01
Case 15	58	F	22	8	1	Positive	82.69	7.95	3.68	51.47
Case 16	61	M	11	14	2	Positive	106.09	15.19	2.12	41.23
Case 17	62	F	1	19	2	Positive	118.69	7.42	11.17	48.09
Case 18	62	F	12	14	2	Positive	70.93	9.53	0.95	59.17
Case 19	63	M	8	11	3	Positive	101.23	15.88	2.83	47.68
Case 20	64	M	9	21	3	Positive	65.93	2.75	0.53	65.80
Case 21	64	F	17	14	1	Positive	87.85	5.30	29.71	81.00
Case 22	66	M	17	9	1	Positive	95.95	4.08	4.05	58.50
Case 23	67	M	25	8	0.5	Positive	72.24	24.17	2.49	74.10
Case 24	68	F	24	8	0.5	Positive	123.44	22.90	3.91	47.30
Case 25	68	M	26	9	0.5	Positive	121.49	14.18	4.54	49.27
Case 26	69	M	21	10	1	Positive	92.25	5.29	20.09	84.22
Case 27	71	M	23	8	0.5	Positive	123.46	12.58	5.36	54.15
Case 28	73	F	22.5	8	0.5	Positive	69.66	10.01	3.63	96.43
Case 29	74	F	19	12	0.5	Positive	97.54	2.96	3.16	70.22
Case 30	74	F	12	10	1	Positive	81.20	19.76	14.57	103.38
Case 31	75	M	16	9	1	Positive	68.02	38.29	3.13	100.97
Case 32	77	M	17	8	0.5	Positive	98.02	8.52	2.11	71.43
Case 33	77	M	20	8	1	Positive	92.65	17.88	2.11	75.09
Case 34	81	F	15.5	13	1	Positive	107.89	7.07	2.08	71.84
Case 35	81	F	14	10	1	Positive	65.07	9.65	4.23	129.74
Case 36	82	M	25	8	0.5	Positive	111.82	31.73	4.27	76.61
Case 37	83	M	12	11	1	Positive	135.02	16.05	3.63	64.04
Case 38	84	F	9	11	2	Positive	86.63	18.33	4.22	104.15

The γ-value is calculated according to the equation: γ = (1/TDP)*([TMP + 1]ˆ[-0.01])*([T + 1]ˆ[1/6])*Ageˆ2 published in our recent study [13].

ADL: Activity of Daily Living; CDR: Clinical Dementia Rating; MMSE: Mini-Mental Status Examination; PiB: Pittsburgh Compound-B; TDP: Thiamine diphosphate; TMP: Thiamine monophosphate.

### The detection of blood thiamine metabolites

The method of HPLC fluoroscopy for measuring thiamine metabolites was essentially the same as previously described [13]. Briefly, 150 µl of whole blood samples anticoagulated with heparin were immediately mixed with 7.4% perchloric acid and then vibrated for 30 s for deproteinization. After being centrifuged at 10,000 rpm and 4°C for 6 min, the supernatants were collected and stored at -20°C and tested within 2 weeks. The samples were protected from light exposure during the whole process. Potassium ferricyanide was added into the supernatants in order to convert thiamine metabolites into thiochromes that were separated by gradient elution by a C18 reversed-phase analytical column (250 × 4.6 mm). The separated thiochromes were then detected by HPLC (Agilent 1100, CA, USA) with an excitation wavelength of 367 nm and an emission wavelength of 435 nm. The levels of blood thiamine diphosphate (TDP), thiamine monophosphate (TMP) and thiamine were quantified according to the standard curve derived from the standard samples of TDP, TMP and thiamine (Sigma-Aldrich, MO, USA).

### ^11^C-PiB synthesis & PET imaging

The radiolabeling synthesis of ^11^C-PiB was based on the method described previously [14]. Approximately 10 mCi radiotracer was injected through the opisthenar vein within 60 s and was flushed with 1 ml saline. Subsequently, a 3D dynamic PET acquisition was performed from 0 to 60 min post injection following an attenuation correction CT using Biograph 64 Truepoint PET/CT scanner (Siemens Medical Solution). The 40–60 min static images were reconstructed using an iterative 3D method with Gaussian filter of 6 mm in Full Width of Half Maximum. The pixel size was 2.0 mm and the slice thickness was 1.5 mm. ^11^C-PiB PET images were visually assessed as ‘PiB positive’ or ‘PiB negative’ by two experienced experts in PET diagnosis, who were blind to clinical data.

### Statistical analysis

The results were shown as mean ± SEM. GraphPad prism software (version 5.01) and SPSS software (version 18.0; SPSS Inc) were used for statistical analysis. Unpaired t-test was used for the comparisons of blood thiamine metabolites between PiB-positive and PiB-negative patients. The diagnostic cut-off point of blood TDP was set up to 99.48 nmol/l, and γ-value was calculated based on the equation: γ = (1/TDP)*([TMP + 1]ˆ[-0.01])*([T + 1]ˆ[1/6])*Ageˆ2 and its cut-off point for AD diagnosis was set up to 75.97 according to our previous study [13]. Four-fold table analysis was used to calculate the sensitivity and specificity of blood TDP and γ-value for the differentiation of PiB-positive patients and PiB-negative patients. The statistical significance of four-fold table analysis was examined by Fisher’s exact test. p < 0.05 was considered statistically significant.

## Results

### Reduced blood TDP level in PiB-positive patients as compared with PiB-negative patients

**Figure 1.**
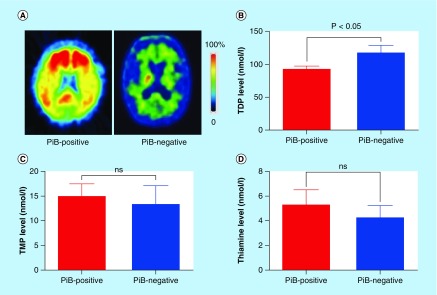
**Altered blood thiamine metabolites in Pittsburgh Compound-B-positive and -negative Alzheimer's disease patients clinically diagnosed.** **(A)** The representative images of ^11^C-PiB PET in PiB-positive and -negative subjects are shown. **(B)** Blood TDP levels were significantly reduced in PiB-positive subjects (n = 33) as compared with that of PiB-negative subjects (n = 5) (p < 0.05). **(C & D)** The levels of blood TMP and thiamine were not significantly different between PiB-positive subjects and PiB-negative subjects (both p > 0.05). PiB: Pittsburgh Compound-B; TDP:Thiamine diphosphate; TMP: Thiamine monophosphate.

There were 33 PiB-positive patients and five PiB-negative patients within the 38 clinically diagnosed AD patients. The representative images of ^11^C-PiB PET in PiB-positive patients showed significant ^11^C-PiB deposition in multiple cortical regions as compared with PiB-negative patients. TDP levels in PiB-positive patients (93.47 ± 3.77 nmol/l, n = 33) were significantly reduced as compared with that in PiB-negative patients (117.80 ± 10.70nmol/l, n = 5; p < 0.05, see Figure 1). There were no significant differences in TMP and thiamine levels between PiB-positive patients (TMP: 15.20 ± 2.26 nmol/l, thiamine: 5.39 ± 1.09 nmol/l) and PiB-negative patients (TMP: 13.61 ± 3.50 nmol/l, thiamine: 4.32 ± 0.92 nmol/l; p > 0.05, Figure 1).

### TDP is a stable & reliable biomarker for AD demonstrated by ^11^C-PiB PET scanning

**Figure 2.**
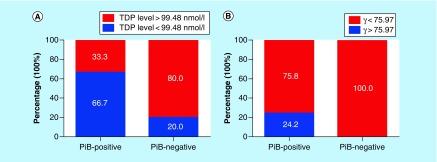
**The diagnostic value of blood thiamine metabolites as a biomarker of Alzheimer’s disease examined by ^11^C-Pittsburgh Compound-B-positive positron emission tomography.** **(A)** The sensitivity and specificity of blood TDP level for the differentiation of C-Pittsburgh Compound-B (PiB)-positive patients and PiB-negative patients demonstrated by ^11^C-PiB positron emission tomography were 66.7% and 80.0% (p = 0.0685), respectively, when the cut-off point was set up to 99.48 nmol/l. **(B)** The sensitivity and specificity of γ-value for the differentiation of PiB-positive patients and PiB-negative patients demonstrated by ^11^C-PiB PET were 24.2 and 100.0% (p = 0.5632), respectively, when the cut-off value of γ-value was set up to 75.97. The variable γ was calculated based on the following equation: γ = (1/TDP)*([TMP + 1]ˆ[-0.01])*([T + 1]ˆ[1/6])*Ageˆ2 published in our previous study [13]. PiB: Pittsburgh Compound-B; TDP: Thiamine diphosphate; TMP: Thiamine monophosphate.

There were 22 of 33 PiB-positive patients (66.7%) with blood TDP levels less than 99.48 nmol/l (the cut-off point), whereas only one of five PiB-negative patients (20.0%) manifested blood TDP levels less than 99.48 nmol/l. Thus, the sensitivity and specificity of TDP for the differentiation of PiB-positive patients and PiB-negative patients demonstrated by ^11^C-PiB PET scanning were 66.7 and 80.0%, respectively (p = 0.0685; Figure 2).

### γ-value did not show high power for AD diagnosis

There were eight of 33 PiB-positive patients (24.2%) with γ-value over 75.97 (the cut-off point) and five of five PiB-negative patients with γ-value less than 75.97. The sensitivity and specificity of γ-value for the differentiation of PiB-positive patients and PiB-negative patients were 24.2 and 100.0%, respectively (p = 0.5632, Figure 2).

## Discussion

Our previous study showed that the reduction of blood TDP and the increase of γ-value representing the best combination of blood thiamine metabolites (TDP, TMP and thiamine) and age displayed significant power for AD diagnosis, with 77.4% of sensitivity and 78.1% of specificity for TDP with the cut-off point set as 99.48 nmol/l, and 81.4% of sensitivity and 90.5% of specificity for γ-value when the cut-off point was set to 75.97 [13]. However, our previous results were derived from the data of subjects of age 65 or above. In this study, we validated the diagnostic value of blood thiamine metabolites in AD cases with a wider age range, including those less than 65 years old. The accuracy of clinical diagnosis of AD (nearly 80%) is still not satisfactory. To confirm the diagnostic value of blood thiamine metabolites in AD, we used ^11^C-PiB PET scanning to enhance the diagnostic accuracy of AD and reexamine the power of blood TDP and γ-value for AD diagnosis.

The results showed that blood TDP levels in 33 PiB-positive patients were significantly reduced as compared with that in five PiB-negative patients. It is consistent with the results of our previous study that blood TDP levels in AD patients were significantly lower than that in control subjects with cognitive abilities in the normal range and patients with vascular dementia and frontotemporal dementia [13]. Further, we found 22/33 (66.7%) of PiB-positive patients with TDP levels under the cut-point of 99.48 nmol/l and 4/5 (80.0%) of PiB-negative patients with TDP levels above the cut-point of 99.48 nmol/l. Thus, the sensitivity and specificity of blood TDP for the differentiation of PiB-positive patients and PiB-negative patients were 66.7 and 80.0% (p = 0.0685), respectively. The findings were consistent with the results of our previous study [13]. However, the exact conclusion needs to be further validated using a larger sample of cases examined by ^11^C-PiB PET, particularly patients with PiB-negative images, in the future studies. Our current study did not show significant performance of γ-value for AD diagnosis. The equation for the calculation of γ-value was established based on 65 years old or above in our previous study [13]. Our current study included AD patients under 65 years. Thus, the previous formula for γ-value calculation may be not suitable for this age group of patients (<65 years old) and a better equation for calculating γ-value without age limitation should be explored in future studies. Overall, owing to the limited sample size of our current preliminary study, we believe that the conclusions we have made require validation in future studies in a larger sample of subjects.

Brain glucose hypometabolism is an invariant feature of AD and closely correlates with cognitive impairment [15,16]. Thus, brain glucose hypometabolism and its associated factor(s) may be potential targets for AD diagnosis and therapy. TDP, a functional thiamine derivative, plays a pivotal role in glucose metabolism as a critical coenzyme of three key enzymes: transketolase, pyruvate dehydrogenatase and α-ketoglutarate dehydrogenatase. TDP level and the enzymatic activities have been demonstrated to be reduced in AD patients [17–20]. The improvement of brain glucose metabolism by nasal insulin can enhance cognitive function and provide neuroprotection on AD patients. Our previous study has illustrated the beneficial effects of benfotiamine, a thiamine derivative, against AD [21]. Future studies should further clarify not only the mechanism(s) of brain glucose hypometabolism and abnormal thiamine metabolism but also their roles as diagnostic and therapeutic targets for AD.

## Conclusion

Our results suggest that blood TDP is a stable biomarker for AD diagnosis while γ-value did not exhibit a significantly diagnostic value for AD, possibly due to the age limitation of participants for the γ-value calculation.

**Executive summary**

- Blood thiamine diphosphate level appears significantly reduced in Alzheimer’s disease (AD) patients.
- Blood thiamine diphosphate appears a stable biomarker for AD diagnosis.
- γ-value did not show high performance for AD diagnosis.
